# Looking Beyond the Core: The Role of Flanking Regions in the Aggregation of Amyloidogenic Peptides and Proteins

**DOI:** 10.3389/fnins.2020.611285

**Published:** 2020-12-01

**Authors:** Sabine M. Ulamec, David J. Brockwell, Sheena E. Radford

**Affiliations:** Astbury Centre for Structural Molecular Biology, School of Molecular and Cellular Biology, Faculty of Biological Sciences, University of Leeds, Leeds, United Kingdom

**Keywords:** flanking region, amyloid, synuclein, fuzzy coat, aggregation, Tau, TDP-43, Orb2

## Abstract

Amyloid proteins are involved in many neurodegenerative disorders such as Alzheimer’s disease [Tau, Amyloid β (Aβ)], Parkinson’s disease [alpha-synuclein (αSyn)], and amyotrophic lateral sclerosis (TDP-43). Driven by the early observation of the presence of ordered structure within amyloid fibrils and the potential to develop inhibitors of their formation, a major goal of the amyloid field has been to elucidate the structure of the amyloid fold at atomic resolution. This has now been achieved for a wide variety of sequences using solid-state NMR, microcrystallography, X-ray fiber diffraction and cryo-electron microscopy. These studies, together with *in silico* methods able to predict aggregation-prone regions (APRs) in protein sequences, have provided a wealth of information about the ordered fibril cores that comprise the amyloid fold. Structural and kinetic analyses have also shown that amyloidogenic proteins often contain less well-ordered sequences outside of the amyloid core (termed here as flanking regions) that modulate function, toxicity and/or aggregation rates. These flanking regions, which often form a dynamically disordered “fuzzy coat” around the fibril core, have been shown to play key parts in the physiological roles of functional amyloids, including the binding of RNA and in phase separation. They are also the mediators of chaperone binding and membrane binding/disruption in toxic amyloid assemblies. Here, we review the role of flanking regions in different proteins spanning both functional amyloid and amyloid in disease, in the context of their role in aggregation, toxicity and cellular (dys)function. Understanding the properties of these regions could provide new opportunities to target disease-related aggregation without disturbing critical biological functions.

## Introduction

In the 60 years since the first atomic structure of the protein myoglobin was solved using X-ray diffraction of protein crystals (Kendrew et al., 1960), the field of structural biology has been dominated by the study of globular proteins with a well-defined tertiary structure, with more than 160,000 unique structures solved to date using crystallography, NMR or cryoEM (Geraets et al., 2020). Despite this feat, more than 50% of the proteins in eukaryotes are now known to have at least one long (>30 residues) sequence that is intrinsically disordered [intrinsically disordered regions (IDRs)] and also 12% of eukaryotic proteins are completely intrinsically disordered [intrinsically disordered proteins (IDPs)] (Dunker et al., 2000; Ward et al., 2004; Tompa, 2009). IDRs and IDPs are enriched in/for proteins with important regulatory or signaling functions (Ward et al., 2004; Tompa et al., 2006; Coletta et al., 2010) demonstrating the crucial role of non-globular protein structures for biological processes.

**FIGURE 1:**
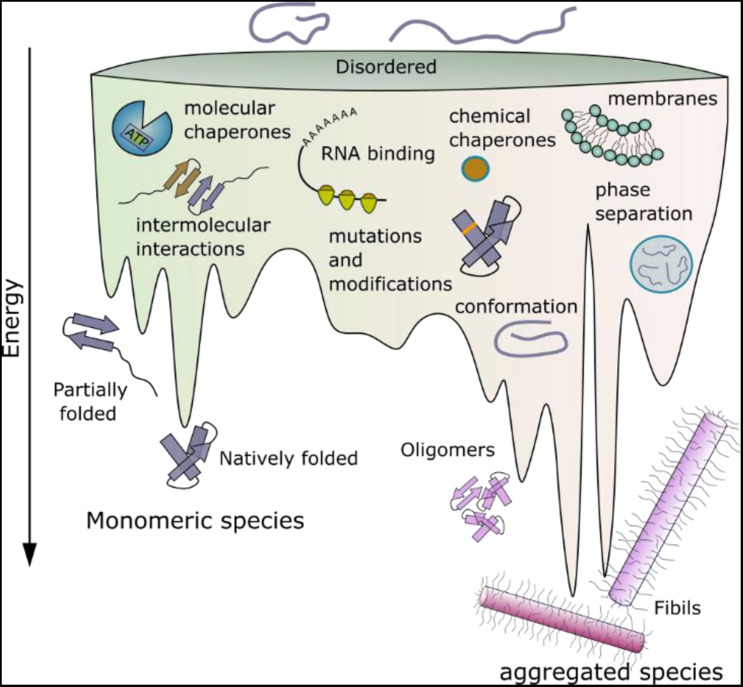
Schematic energy landscape of folding and aggregation of proteins focusing on factors affecting the kinetics of folding and amyloid assembly. The surface shows the conformations that proteins can adopt (monomeric and aggregated species) during folding and aggregation into amyloid. The lowest energy state is the amyloid fibril (Baldwin et al., 2011). Adopting a folded native structure or stabilization of this structure by ligand binding, disfavors amyloid formation, whilst mutations or post-translational modifications that favor inter-molecular interactions and phase separation can destabilize the monomer and tip the balance toward fibril formation. Binding of a protein to a membrane surface can also favor aggregation, by changing the structure of the amyloid precursor and driving inter-molecular interactions. Note that the relative position of each folding- and aggregation-factor within the energy landscape is arbitrary, as these factors can influence folding and aggregation at multiple different points. Their relative position, therefore, does not imply that they act at only one point on the landscape, or interact with a specific folding or aggregation intermediate.

Amyloid proteins, some of which are involved in neurodegenerative disorders such as Alzheimer’s disease, Parkinson’s disease, amyotrophic lateral sclerosis, and Huntington disease (Iadanza et al., 2018a; Benson et al., in press) are commonly IDPs or are proteins that contain IDRs. These diseases share a common fundamental etiology: aberrant self-assembly of their amyloid precursor proteins to form toxic oligomers and highly ordered fibrils with a cross β-sheet structure (Gallardo et al., 2020b; Ulamec and Radford, 2020). A combination of biochemical and biophysical approaches, including limited proteolysis, hydrogen exchange (HX), solution NMR, solid state NMR (ssNMR), cryoEM and EPR have shown that the structured cross-β amyloid core commonly involves only a portion of the amyloid precursor sequence, whilst regions flanking the fibril core (commonly the N- and/or C-terminal regions of the sequence), are flexible and thus are either “invisible” in the structures determined or give rise to only low resolution density in EM images (Gallardo et al., 2020b). Consequently, the high resolution cryoEM and ssNMR structures of fibril architectures determined over the last ∼5 years, have necessarily focused on the conformations and interactions within fibril cores (Gallardo et al., 2020b), whilst the more dynamic flanking regions remain elusive. The structured cores of amyloid fibrils usually contain short peptide sequences, some with high aggregation propensity, shown to be necessary and sufficient for fibril formation (Giasson et al., 2001; Thompson et al., 2006) and reviewed in Eisenberg and Sawaya (2017). Despite the importance of these sequences for the formation and stability of the amyloid fold, several studies have shown that modifying or changing (e.g., by deletion, mutation, or post-translational modification) the regions that flank the amyloid core can affect the fibril growth kinetics (Johnson et al., 2009; Benilova et al., 2014; Doherty et al., 2020), fibril morphology (Guerrero-Ferreira et al., 2020; Scheres et al., 2020) and the formation of crucial contacts with interaction partners (Gao et al., 2015; Burmann et al., 2020; Figure 1). Additionally, the presence of so called “gatekeeper” residues, which surround aggregation-prone regions (APRs) to mitigate their aggregation propensity (Reumers et al., 2009) further underlines the important role of residues not directly involved in forming the amyloid core in the process of aggregation and in the functional consequences of the fibril structure formed. The aim of this review is to highlight the importance of the dynamically disordered flanking regions in amyloid sequences, focusing on their roles in fibril formation, cytotoxicity, and other physiological functions. While more than 50 proteins are known to form amyloid associated with disease (Iadanza et al., 2018a; Benson et al., in press), here we focus on αSyn, TDP-43, Aβ, Tau, β_2_m, Orb2, and PrP as exemplars of IDPs, natively folded proteins, prions, and functional and disease related proteins.

## Flanking Regions – What Are They and Why Are They Important?

**FIGURE 2:**
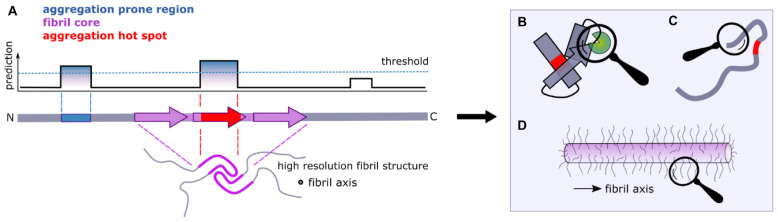
Flanking regions and amyloid formation. **(A)** Computational approaches can be used to identify aggregation-prone regions (APRs) from sequence data alone (blue). Fibril cores are defined by cryoEM and ssNMR structures of amyloid fibrils and include residues that adopt a stable cross-β structure (purple). When the APRs and the fibril core overlap, we term the region as an “aggregation hotspot” (red). Large segments of a protein sequence can remain dynamically disordered in amyloid structures (lower image). **(B)** Interaction partners and/or **(C)** the formation of transient non-local intra-molecular interactions that involve regions that flank the APRs can have significant effects on the aggregation kinetics of amyloidogenic proteins, as well as on their function. **(D)** Dynamically disordered regions flanking the amyloid core in the fibrillar state form an amyloid “fuzzy coat” that can impart biological functions to the amyloid fold.

Decades of studies of protein folding have led to insights into the roles of individual amino acids in a protein sequence in the search for the native fold (they are kinetically important) and in stabilizing the final folded state (they are thermodynamically important) (Rumbley et al., 2001; Baldwin et al., 2011). In addition, some residues may be conserved because they are important in chaperone binding or in the destabilization of incorrect folds (so-called negative design) (Richardson and Richardson, 2002; Berezovsky et al., 2007). By contrast with our the wealth of knowledge about protein folding, predicting which residues in an amyloid precursor sequence could be kinetically important in driving or controlling the rate or mechanism of amyloid fibril formation; which form the stable fibril core; and which may be innocuous passengers during self-assembly, but may play a role in the fibrillar state, is immensely complex. This is a fundamentally important question, since it is now widely appreciated that, by contrast with protein folding wherein the same globular structure is (usually) adopted by a protein sequence independent of mutation or changes in the solution conditions, amyloid fibril formation is under kinetic control, with the structure of the fibril product being determined by the assembly pathway taken (Figure 1). The result is a rugged energy landscape, which can result in potentially many different fibril structures for the same (or very similar) protein sequences (known as fibril polymorphism) (Fitzpatrick et al., 2017; Close et al., 2018; Kollmer et al., 2019; Guerrero-Ferreira et al., 2020). Recent advances in computational methods have provided a suite of algorithms able to define the most aggregation prone region of a protein sequence (Fernandez-Escamilla et al., 2004; Sormanni et al., 2015; Ebo et al., 2020; Figure 2A). These regions are commonly found in the amyloid fibril core, stabilizing the final fibril structure, without necessarily playing a role in the kinetics of amyloid formation. Other residues in amyloidogenic peptides and proteins which flank the APRs, can play kinetic and/or thermodynamic roles in amyloid assembly. Changes in these sequences (mutation or post-translational modification) can dramatically alter the rates of fibril formation and the structures of the fibrils formed, without the sequence necessarily forming part of the stable fibril core. As more fibril structures are solved of full-length protein sequences (rather than short peptide fragments), it is becoming clear that the amyloid core can involve only a minor part of the protein sequence, with substantial regions of the polypeptide chain remaining dynamically disordered in the fibril structure (Figure 2A). Such regions which flank the amyloid core can play functional roles (binding ligands and receptors for functional amyloid), or perturbing proteostasis (for amyloid fibrils associated with disease) (Figures 2B–D).

**FIGURE 3:**
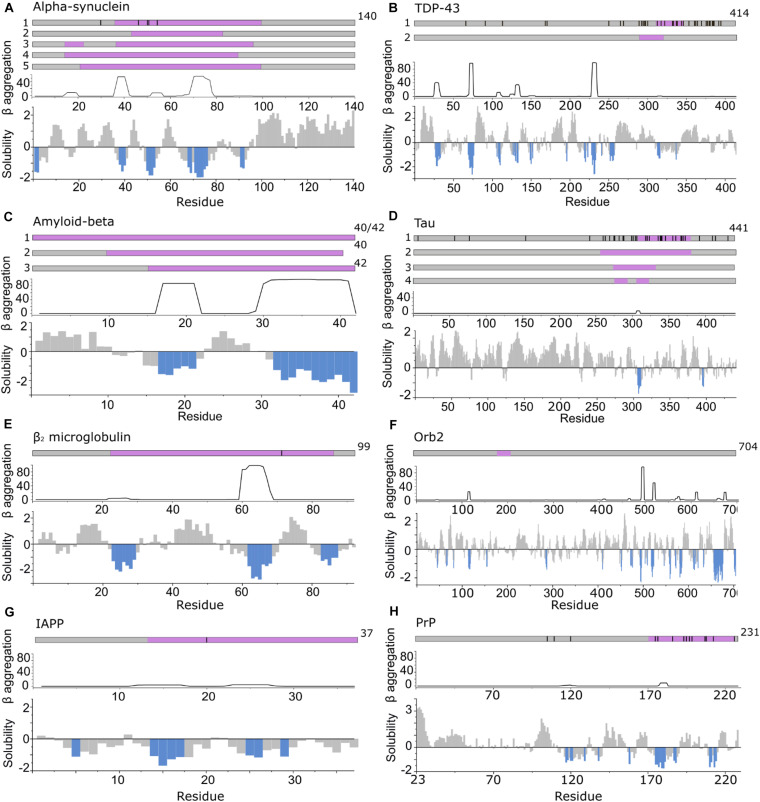
APRs comprise only a small part of the amyloid core. **Top in A–H:** location of fibril cores of αSyn, TDP-43, Aβ, Tau, β_2_m, Orb2B, IAPP, and PrP defined by recent cryoEM or ssNMR fibril structures (purple). The positions of familial disease mutations are highlighted where appropriate as black lines. **Bottom in A–H:** regions with low solubility predicted by CamSol (below –1 is aggregation promoting highlighted in blue) (Sormanni et al., 2015) and the β-aggregation potential of each sequence predicted using TANGO (Fernandez-Escamilla et al., 2004). **(A)** αSyn including polymorph 1a (1) (Guerrero-Ferreira et al., 2018; Li B. et al., 2018; Li Y. et al., 2018) (core residues 37–99), 1b (2) (Li B. et al., 2018) (core residues 43–83), 2a and b (3) (Guerrero-Ferreira et al., 2019) (core residues 14–24, 36–96) and the MSA *ex vivo* structures including residues 14–94 (for PF-IA and PF-IIA) (4) or residues 21–99 (for PF-IB and PF-IIB) (5) in the fibril core (Schweighauser et al., 2020). **(B)** TDP-43 cryoEM structure solved from C-terminal segments forming a dagger shaped core (1) (residues 312–346) or R-shaped core (2) (residues 288–319) (Cao et al., 2019). **(C)** Aβ structures solved (1) for Aβ_42_ (Gremer et al., 2017) and Aβ_40_ in which all residues comprise the core (Lu et al., 2013; Kollmer et al., 2019) (2) fibrils in which the core is formed by residues 10–40 for Aβ_40_ (including polymorphs 2A and 3Q) (Petkova et al., 2002; Paravastu et al., 2008) and (3) for Aβ_42_ (core formed by residues 15–42) (Colvin et al., 2016; Wälti et al., 2016). **(D)** Tau fibril structures PHF and SF from Alzheimer disease patients (1) (Fitzpatrick et al., 2017) (core residues 306–378), NPF and WPF from Pick’s Disease (2) (Falcon et al., 2018) (core residues 254–378) and heparin induced structures 4R-s and 3R formed *in vitro* (3) (core residues 272–330) and 4R-t and 4R-j (4) (core residues 274–292, 304–321, respectively) (Zhang et al., 2019). **(E)** The β_2_m fibril core involves residues 22–85 (Iadanza et al., 2018b; Gallardo et al., 2020a). **(F)** The Orb2B fibril core consists of residues 176–206 (Hervas et al., 2020). **(G)** Human IAPP forms fibrils with residues 13–37 (Röder et al., 2020), 14–37 (Cao et al., 2020), or 13–37 (Gallardo et al., 2020a), with its early onset S20G variant adopting fibrils with two- and three filaments involving residues 15–37 in the core (Gallardo et al., 2020a). **(H)** PrP fibrils form fibril core with residues 170–229 revealed using cryoEM (Wang et al., 2020).

In this review we focus on the roles of regions that flank the APRs and/or fibril cores in amyloid formation. We differentiate between (i) sequences that flank the APRs determined by *in silico* techniques, (ii) sequences that flank the structured cross-β fibril core based on recent cryoEM and NMR structures of fibrils, and (iii) sequences that flank coincident APRs and fibril cores (producing an “aggregation hotspot”) (Figure 2). In 2005 used a similar approach where they differentiated between regions that are predicted to be most important for promoting amyloid growth and experimentally determined sensitive regions Dobson and coworkers (Pawar et al., 2005). It is important to note that different flanking regions can be identified in the same protein using these definitions. For example, the use of different algorithms [e.g., CamSol or TANGO (Figure 3)] as well as posttranslational modifications, might result in different flanking regions for the same protein primary sequence. Similarly, on a structural level, as polymorphs for the same protein have different residues involved in their cores, each will have a different flanking region (Figure 3).

## Identification of Sequences Involved in Forming Amyloid

Prior to the development of high-resolution structural methods capable of solving amyloid structures in atomic detail, lower resolution techniques were employed to provide information about the sequences that drive aggregation. These include analysis of the ability of arrays of peptide fragments from different amyloid precursor sequences to form cross-β amyloid-like structures in isolation (Tenidis et al., 2000; von Bergen et al., 2000; Jones et al., 2003a; Ivanova et al., 2004; Nelson et al., 2005), scanning mutagenesis of a sequence followed by analysis of fibril formation using aggregation assays (e.g., Thioflavin T fluorescence) (Williams et al., 2006; Platt et al., 2008), and determination of the sequences that form the stable amyloid core, e.g., using protease digestion followed by mass spectrometry (Miake et al., 2002; Myers et al., 2006; Kushnirov et al., 2020) or HX monitored by ^1^H-NMR (Hoshino et al., 2002; Cho et al., 2011; Strohäker et al., 2019). These experimental approaches have been complemented by the development of *in silico* tools (Ebo et al., 2020; Santos et al., 2020), able to identify APRs by calculation of β-sheet propensity (using TANGO) (Fernandez-Escamilla et al., 2004) or solubility (CamSol) (Sormanni et al., 2015) (see Santos et al., 2020 for a recent review of these and other approaches). Most, but not all APRs are found in the fibril core. Residues that are found in fibril cores determined by cryoEM or ssNMR and in the APRs identified using computational methods described above are here defined as “aggregation hotspots” (Figure 3). They often contain motifs crucial for fibril formation [e.g., the Non-Amyloid β-Component (NAC) region of αSyn, the ^22^NFGAIL^27^ sequence from human islet amyloid polypeptide (IAPP), ^15^KLVFF^20^ for Aβ and ^306^VQIVYK^311^ from Tau].

Huge strides have been made in amyloid fibril structure elucidation in the last decade using X-ray diffraction of microcrystals (usually of short, 6–15 residue peptides) (Rodriguez et al., 2015; Guenther et al., 2018), and, more recently, using ssNMR and cryoEM of amyloid fibrils formed *in vitro* and *ex vivo* from full-length proteins. These studies have shown that the same sequence can produce fibrils with remarkably different quaternary, tertiary and even secondary structural elements (Gallardo et al., 2020b). For example, more than six different amyloid fibril structures of the 140 residue protein, α-synuclein (αSyn) have been solved to date using cryoEM or ssNMR (Table 1; Guerrero-Ferreira et al., 2020; Schweighauser et al., 2020). Notably, while the fibril cores in all of these structures contain the NAC region known to be necessary and sufficient for amyloid formation (Giasson et al., 2001), the length and location of the sequence involved in the remaining portions of structured amyloid core (or conversely the residues involved in unresolved, dynamically disordered regions) varies depending on the morphology of the amyloid fibril formed (Figure 3; Guerrero-Ferreira et al., 2019, 2020; Schweighauser et al., 2020). Thus, between 50% and 70% of the 140 residues of this protein are *not* involved in the cross-β amyloid core. A second striking example is the two fibril structures formed from antibody light chains (LCs) that were extracted from two patients with systemic LC amyloidosis (involving different LC sequences). These studies revealed that these proteins with the same initial immunoglobulin (Ig)-containing native structure (Huang et al., 1996; Swuec et al., 2019), form completely different amyloid fibril architectures, with residues 16–23 and 86–93 (Radamaker et al., 2019), or residues 1–37 and 66–105 forming the core (Swuec et al., 2019).

**TABLE 1:** Summary of the functional roles of different regions of the amyloid proteins shown in Figure 3.

Protein	Residues/region	(Dys-)function	References
αSyn			
	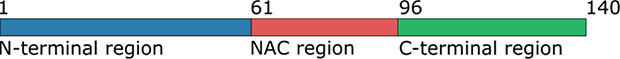		
	1–14	Membrane insertion	Cholak et al., 2020
	1–25	Initial membrane binding	Fusco et al., 2014
	Extreme N-terminus and region around Y39	Chaperone binding	Burmann et al., 2020
	36–42 + 45–57	Involved in liposome clustering	Doherty et al., 2020
	36–42 + 45–57	Forms intra- and intermolecular interactions important for fibril formation	Doherty et al., 2020
	37–54	Forms β-hairpin crucial for nucleation/oligomerization processes	Mirecka et al., 2014
	C-terminal region (91–140)	Protects protein from aggregation by shielding NAC region and/or β-hairpin C-terminal truncation (109-140) results in faster aggregation	Hoyer et al., 2004; Hong et al., 2011; Yu et al., 2015; Stephens et al., 2019
	C-terminal region (residues 110–140)	Binding to chaperone-like protein SERF accelerates aggregation	Falsone et al., 2012
	C-terminal region (residue 125–129)	Dopamine binding drives off-pathway oligomer formation	Herrera et al., 2008

TDP-43			
	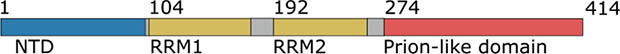		
	3–183	Interactions initiate homodimerization important for polymerization dependent splicing activity	Shiina et al., 2010; Afroz et al., 2017
	1–10 (especially Arg6, Val7, Thr8, and Glu9)	Mediates full-length TDP-43 oligomerization important for splicing activity and key to initiate aggregate formation	Zhang et al., 2013
	RRM1 (104–176), especially residue I107, D105, L111, W113, Q134, G146, F147, F149, R171, K176, N179 [RRM2 (192–262)]	Binds TG-rich DNA and UG-rich RNA for function (e.g., splicing, translation control, transport). RRM2 shows lower binding affinity.	Lukavsky et al., 2013; Kuo et al., 2014
	RRMI1 (residue F147 and F149) and residue 208–441	Prevents aggregation by enhancing solubility when bound to single stranded RNA/DNA	Huang et al., 2013
	RRM1 (residue F147 and F149) and 321–366	Autoregulation of own protein expression by binding to its mRNA	Ayala et al., 2011
	Residue 320–340, especially W334, W385, and W412	Involved in liquid-liquid phase separation	Conicella et al., 2016; Sun and Chakrabartty, 2017; Li H.R. et al., 2018

Aβ			
	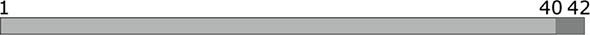		
	N-terminal domain (residues 1–17)	Binding to cystatin C (cysteine protease inhibitor)	Sastre et al., 2004
	Aβ_40_: central region (residues 25–29); part of the structured fibril core but solvent accessible	Disaggregase activity when binding Lipocalin-type Prostaglandin D synthase (L-PGDS)	Kannaian et al., 2019

Tau			
	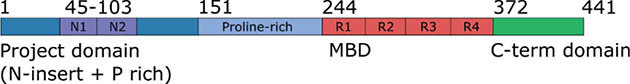		
	1–202	Binding to plasma membrane	Brandt et al., 1995
	N-terminal domain (1–150) interacts with proline rich domain (151–244)	Dimerization (head to tail), suggested to be the natural form for function and toxicity	Rosenberg et al., 2008
	Residue 1–117 and 118–402	Electrostatic interactions between these regions drive phase separation	Boyko et al., 2019
	114–193 (P-rich domain) and 198–278 (microtubule-binding domain)	Actin binding and promoting F-actin bundling and G-actin assembling	He et al., 2009
	N-terminal domain, proline-rich region and MBD	Chaperone binding	Mok et al., 2018
	Proline rich domain, MBD	Interaction and polymerization of tubulin	Barbier et al., 2019; Chen et al., 2019; McKibben and Rhoades, 2019
	Proline rich domain and C-terminal domain	Main locations of phosphorylation sites, but can be found throughout the whole sequence	Liu et al., 2007
	MBD (295–305)	β-hairpin formation that protects the aggregation prone 306–311 region	Chen et al., 2019
	MBD (residue 275–280 and 306–311) and other regions	Heparin binding drives aggregation; MBD shows highest affinity to heparin	Sibille et al., 2006

β_2_m			
	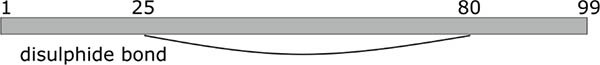		
	Residue 1–6	Stabilization of native structure; accelerates aggregation when deleted	Esposito et al., 2000
	A and G strand (I7A, V9A, and V93A)	Mutations drive fibril growth by destabilizing local tertiary structure and increasing dynamics	Jones et al., 2003b
	A, B, E, F strand (6–11, 21–28, 64–70, 79–83)	Interaction with chaperone αB-crystalline preventing oligomerization and fibril formation	Esposito et al., 2013

Orb2B			
	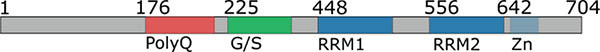		
	RNA binding domain	Interaction with RNA facilitates long term memory formation	Krüttner et al., 2012

IAPP			
	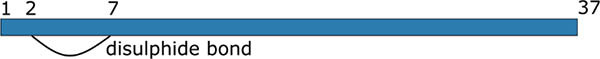		
	1–19	Membrane binding and disruption	Brender et al., 2008
	1–17 and/or 30–37	Liquid-liquid phase separation	Pytowski et al., 2020

PrP			
	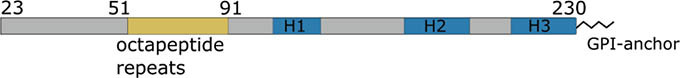		
	N-terminal region (residues 23–90)	Interaction with Tau	Han et al., 2006
	N-terminal region (residue 23–89)	Interaction with αSyn fibrils facilitating αSyn cell-to-cell spreading	Aulić et al., 2017
	Residues 95–110	Receptor binding site for Aβ42-oligomers	Laurén et al., 2009
	Hydrophobic region (residue 111–134)	Hydrophobically driven binding/insertion with anionic membranes, this interaction is important for (murine) PrP to gain C-terminal Proteinase K resistance and convert it to PrP^Sc^	Wang et al., 2010b
	Octapeptide region in N-terminal domain	Increased numbers of octapeptides that bind Ca^2+^ promotes fibril formation and disease development	Goldfarb et al., 1991

*Each protein sequence is coloured, highlighting regions with different functional activity or those which have been mapped biochemically. RRM, RNA recognition motif; MBD, microtubule binding domain.*

In general, *in silico*-identified APRs (which may differ slightly when using different algorithms) are shorter in length compared with the experimentally determined fibril core using cryoEM or ssNMR (Figure 3). It should be noted that *in silico* methods also identify APRs that can reside outside the structured amyloid core, and so form the flanking region of aggregation hotspots (Figure 2A). A particularly striking example of this is seen in the functional amyloid Orb2, associated with long term memory formation and storage (Keleman et al., 2007; Majumdar et al., 2012; Khan et al., 2015; Hervas et al., 2020), where there is no overlap of sequence between residues in the experimentally derived fibril core and those in APRs identified using *in silico* methods (Figure 3F). This clearly demonstrates the division of sequence motifs into those which may be kinetically important, those which are thermodynamically important, and some which play neither or both roles.

Similar to the diversity of amyloid flanking regions, it can also be difficult to parse structured and unstructured regions. For the functional amyloid protein Sup35, for example, which is involved in controlling translation in yeast (Lyke et al., 2019), site-directed mutagenesis and fluorescence labeling suggested that the amyloid core comprises amino acids 21–121, flanked by a structurally heterogeneous “transition zone” (residues 1–20 and 122–158), and a very flexible C-terminal region formed by residues 159–250 (Krishnan and Lindquist, 2005). Another example of different residual mobility of flanking regions was seen by performing immunogold labeling and transmission EM, force-volume measurements using atomic force microscopy (AFM) and solution NMR experiments on Tau filaments (Sillen et al., 2005; Wegmann et al., 2013). These experiments suggested that the flanking regions of Tau form a “two layered polyelectrolyte brush” surrounding the fibril core: a dense and mechanically more rigid layer (residues ∼173–243) and an N-terminal, less dense and more dynamic layer (residues ∼1–172). The increased exposure to solvent renders longer unstructured regions, such as those in Tau and αSyn sensitive to environmental conditions: Tau amyloid fibrils exhibit a 50% reduction in the rigidity of flanking regions upon increasing KCl concentration from 50 mM to 200 mM and show increased affinity (70% higher adhesion) to negatively charged membranes when lowering the pH from 7.4 to 4.5 (Wegmann et al., 2013). These changes may be relevant during lysosomal degradation (Wang et al., 2009). Finally, while not part of the structured β-sheet rich fibril core, some sequences in the flanking regions can nonetheless form secondary structure as shown using ssNMR for the 17 amino acid long N-terminal region (residues 4–11) of exon 1 of huntingtin. In this case, the region flanking the aggregation-prone expanded polyQ region adopts a solvent exposed and dynamic α-helical structure (Sivanandam et al., 2011).

## Role of Flanking Regions and Aggregation Hotspots in Fibril Formation

**FIGURE 4:**
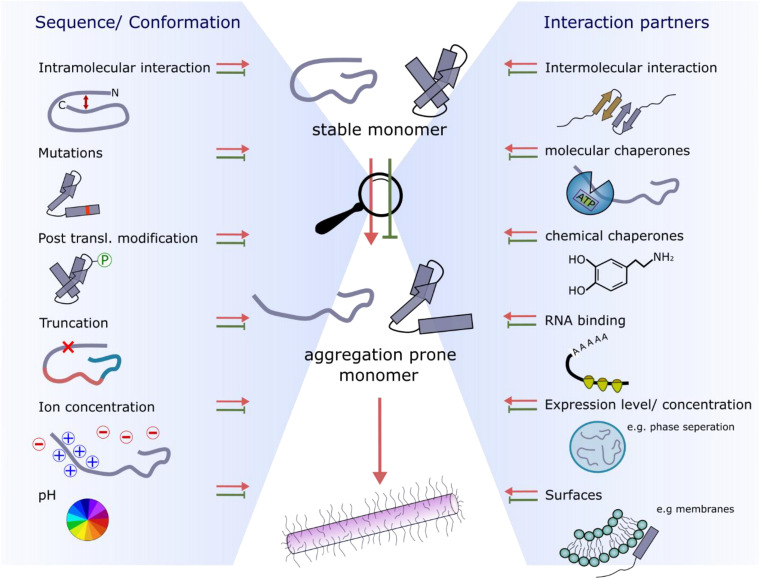
Factors affecting the conformational properties and interactions with amyloid precursors that retard or accelerate fibril growth. The two main influences are sequence/conformation-dependent **(left-hand side)** or occur as a result of binding to interaction partners **(right-hand side)**.

Flanking regions have been shown to play roles in modulating the rates and mechanisms of aggregation (Benilova et al., 2014; Doherty et al., 2020). For example, point mutations linked with early onset familial Parkinson’s disease (Mehra et al., 2019) and ALS (Prasad et al., 2019) or post-translational modifications (including phosphorylation, acetylation, sumoylation, methylation, ubiquitination, glycosylation, and truncations that alter aggregation kinetics) are often remote from the APRs within these protein sequences (Figures 3A,B). These regions also bind to other molecules such as chaperones, nucleic acids, and membranes, and hence, can play functionally important roles (Table 1). Consequently, these regions re-sculpt the aggregation energy landscape (Figure 1) enhancing [or in some cases suppressing (Jonsson et al., 2012)] aggregation and its associated cytotoxicity, and can alter the function of the native amyloid precursor. It is thus crucial to analyze these regions when assessing amyloid formation mechanisms, rather than focusing solely on short peptide sequences that constitute the APRs. The latter approach, however, can be incredibly fruitful, leading to novel anti-microbial agents (Khodaparast et al., 2018) and potential cancer treatments (Gallardo et al., 2016). The latter study showed that a *de novo* designed peptide, vascin, based on an amyloidogenic fragment of vascular endothelial growth factor receptor 2 (VEGFR2) knocked down VEGFR2 as a consequence of VEGFR2-dependent fibril growth (Gallardo et al., 2016). Importantly, aggregation kinetics can be either slowed or accelerated depending on the protein and/or the precise modification of the flanking sequences. This is particularly apposite for IDPs, as the shallow, but rough, energy landscapes of these proteins renders their conformational ensemble sensitive to changes in their sequence and environment. This can result in APRs or binding motifs being exposed or sequestered, which in turn can accelerate, slow or prevent aggregation for the same protein sequence relative to a reference condition. Changes in pH, ionic strength and even being in a different cellular environment (e.g., oligodendrocytes vs neurons) can thus have a significant effect on fibril formation as shown for αSyn (Peng et al., 2018; Stephens et al., 2019). Indeed, far from being passive bystanders, flanking regions may be as important in defining the physiological role and amyloid disease etiology as the canonical APRs themselves (Figure 4). Here we highlight examples where flanking regions are involved in promoting or disfavoring aggregation to draw an overview of the significance of sequence and interaction partners of amyloidogenic proteins and peptides for fibril formation.

### Flanking Regions That Protect Against Aggregation

A protein concentration higher than the critical amyloid concentration (Xue et al., 2008), and (sometimes) the presence of surfaces that can act as nucleation sites are required to overcome the energy barrier needed to form amyloid structures (Buell, 2017). Such surfaces include membranes, other proteins, or the air-water interface [so-called heterogeneous primary nucleation (Buell, 2017; Figure 4)] or the amyloid fibril surface itself (secondary nucleation) (Linse, 2017). In the crowded cell, there is a fine-balance of expressing sufficient protein to maintain function, but avoiding high expression levels to disfavor aggregation. This strategy was described as “life on the edge” by Dobson and co-workers (Tartaglia et al., 2007). Interestingly, more recent work has shown that regions surrounding an aggregation hotspot can play a role in maintaining the correct balance between expression level and aggregation potential, such that aggregation is disfavored, whilst the function of the native protein is maintained. A particularly clear example is the TAR DNA-binding protein 43 (TDP-43). The formation of a negative feedback loop involving the RNA-recognition motif 1 (RRM1, especially residues F147 and F149) and the fibril core-forming C-terminal region [residues 321–366 (Figure 3B)] self-regulates protein expression via a mechanism in which TDP-43 binds its own mRNA leading to decreased protein expression (Ayala et al., 2011; Table 1). Single stranded RNA (ssRNA) or DNA (ssDNA) binding to the flanking regions of TDP-43 also functions as a protective mechanism against amyloid formation by increasing the solubility of TDP-43 (Huang et al., 2013). In addition to translational control, the flanking regions of TDP-43 have also been found to affect its aggregation kinetics directly. For example, cells overexpressing Δ1–10 TDP-43 do not form cellular inclusions, by contrast with cells producing the full-length protein (Zhang et al., 2013). Another example where flanking regions protect from aggregation is the homo-tetrameric protein transthyretin (TTR), which is expressed in the liver and cerebrospinal fluid and is associated with amyloidosis. For this protein, the gatekeeper residue K35 [located just at the edge of the fibril core of a recently solved cryoEM structure (Schmidt et al., 2019)] protects the full-length protein from fibril formation (Sant’Anna et al., 2014).

Flanking regions can also limit fibril formation by binding to chaperones, which stabilize and protect the native state of the protein (Wentink et al., 2019; Figure 4). Using *in vitro* and in cell NMR Burmann et al. (2020) recently showed that αSyn is bound to an array of molecular chaperones. Some of these are physiologically relevant (e.g., hsc70, hsp90) whereas others are not (e.g., the periplasmic bacterial chaperones Skp and SurA). Similarly, the ATP-independent nascent polypeptide associated complex (NAC) has been shown to bind to the flanking regions of αSyn and ataxin 3 and retard their aggregation (Martin et al., 2018; Shen et al., 2019). Despite their diversity, all six chaperones in the study by Burmann et al. (2020) were found to bind the N-terminal 10 residues of αSyn and a segment around residue Y39 (Table 1). Inhibition of chaperones Hsc70 and Hsp90 in mammalian cells resulted in the re-localization of αSyn from the cytosol to the mitochondria, amplified fibril growth and increased membrane binding, which has been shown to be the toxic form of αSyn in yeast (Newberry et al., 2020). Interestingly, phosphorylation of Y39, which is associated with αSyn-dependent neurodegeneration (Brahmachari et al., 2016), decreased the interaction with chaperones. Enhancing the concentration of various heat shock proteins thus disfavors αSyn aggregation, potentially providing a therapeutic approach to treat αSyn-aggregation and its associated cytotoxicity (Jones et al., 2014). Similar aggregation-suppressive effects have been reported for other chaperone:protein pairs: β_2_-microglobulin (β_2_m) interacts with αB-crystallin mainly via its flanking regions [residues 6–11, 21–28, 9–83 (Figure 3E and Table 1)] and some residues within the APR (residues 64–70) (Esposito et al., 2013); the N-terminal domain of Tau (residues ∼1–40) (as well as the proline-rich region and repetition motifs [residues ∼160–370 (Figure 3D and Table 1)] binds to the chaperone DnaJA2 (Mok et al., 2018) and the N-terminal 17 residue flanking region of the huntingtin protein forms a complex with the molecular chaperones Hsc70 (Monsellier et al., 2015) or TriC (Tam et al., 2009) reducing its aggregation. This might be explained by the fact that chaperones bind to flanking regions preventing the formation of crucial conformations/interactions required for amyloid formation. For instance, the exon 1 domain of huntingtin has been shown to initiate aggregation by dimerization of its N-terminal 17 residues (Kelley et al., 2009) which is inhibited by chaperone binding to this sequence (Tam et al., 2009; Monsellier et al., 2015).

By contrast with the chaperones discussed above, other chaperone-like proteins have been shown to accelerate aggregation. For example, the binding of the human protein SERF (or MOAG-4, the SERF homolog in *Caenorhabditis elegans*) has been shown to accelerate aggregation of polyglutamine peptides, huntingtin, Aβ and αSyn *in vitro* and *in vivo* (van Ham et al., 2010; Falsone et al., 2012; Meinen et al., 2019). Although the binding site on SERF for all of these amyloidogenic proteins has yet to be identified, αSyn was shown to bind via its C-terminal region (residues 110–140) (Falsone et al., 2012). In other cases, binding of small molecules [chemical chaperones (Figure 4)] has been shown to accelerate or to retard the rate of aggregation and/or the morphology of the fibrils formed. For example, the glycosaminoglycan heparin, has been shown to induce the aggregation of Tau, resulting in fibrils with a different structure from Tau fibrils extracted from the brains of patients with Alzheimer’s, Pick’s or other Tau-associated neurodegenerative disorders (Zhang et al., 2019; Scheres et al., 2020; Figure 3D). NMR studies revealed multiple binding sites on Tau for heparin, with binding affinities ranging from 10 μM to the mM range. The tightest binding is observed within the microtubule binding domain (MBD) (residues 275–280 or 306–311), again highlighting that binding outside the APR modulates aggregation (Sibille et al., 2006). As a final example, the small molecule dopamine binds the C-terminal region of αSyn (residues 125–129), driving off-pathway oligomer formation that does not result in fibril growth (Herrera et al., 2008).

### Flanking Regions That Accelerate Aggregation

As several amyloidogenic proteins are IDPs, transient intra- or inter-molecular interactions mediated by flanking regions can play an important role in defining the overall aggregation propensity of a protein sequence by altering the solvent accessibility of key APRs (Figure 4). For example, paramagnetic relaxation enhancement (PRE) NMR and computational modeling experiments revealed that the flexible negatively charged C-terminal region of αSyn (residues 96–140) forms intra- and inter-molecular interactions with the positively charged N-terminal region of the protein, which protect the aggregation-prone NAC region (Hong et al., 2011; Janowska et al., 2015; Yu et al., 2015; Stephens et al., 2019; Table 1). Conditions that disfavor these transient interactions [e.g., high cation concentration (Nath et al., 2011), low pH (Hoyer et al., 2002) or familial Parkinson’s disease mutations (Ranjan and Kumar, 2017)] result in perturbation of the protective long-range contacts and accelerate aggregation (reviewed by Stephens et al., 2019). Likewise, truncation of the protective C-terminal region of αSyn, as found in Lewy bodies in disease-associated brains (Muntané et al., 2012), causes more rapid fibril formation, potentially rationalizing the role of truncation of these regions in the development of disease (Hoyer et al., 2004). Similarly, for TTR the C and D strands (not part of the aggregation hotspot) are involved in the interchain contacts that lead to aggregation (Kelly and Lansbury, 1994; Palaninathan et al., 2008). Also, many disease associated mutations of TTR (e.g., V30M and L55P) are located within the C and D strand region (Murakami et al., 1992; Lashuel et al., 1999).

More recently, studies analyzing the aggregation kinetics of N-terminal deletion variants of αSyn have shown that specific regions of the protein are also required for the aggregation of full-length αSyn, building on an array of previous data that suggested an importance of the N-terminal region of the protein for its function (membrane binding) (Fusco et al., 2016; Cholak et al., 2020) and its aggregation (Kessler et al., 2003; Terada et al., 2018). Perhaps most remarkably, based on prediction of aggregation-prone and insoluble regions (Figure 3A) discrete sequences were identified that form a range of precise interactions with residues in the NAC and C-terminal regions, protecting the protein from aggregation (Doherty et al., 2020). Deleting residues 38–61 (that encapsulates a region (residues 47–56) shown by Eisenberg and Sawaya (2017), to form fibrils in isolation and named the pre-NAC region (Rodriguez et al., 2015), or deleting/replacing an even shorter peptide [named P1, residues 36–42] or P1P2 (residues 36–57) results in significantly slower fibril growth compared with the wild-type protein both *in vitro* and in *C. elegans* models (Doherty et al., 2020). The reduced aggregation rate may be a consequence of preventing the formation of inter-molecular contacts between regions of the IDP that form a transient β-hairpin structure (strand 1: residue 37–43 and strand 2: residue 48–54) previously postulated based on Thioflavin T assays and molecular dynamics simulations to drive aggregation (Mirecka et al., 2014; Yu et al., 2015). In accord with this hypothesis, binding of a nanobody known as a β-wrapin to this motif prevents aggregation *in vitro* and in Drosophila (Mirecka et al., 2014; Agerschou et al., 2019). Similarly, a specific short aggregation-modulating peptide sequence that lies outside its APRs has been observed for another IDP, Tau, but here the formation of a β-hairpin structure involving residues 295–311 protects the aggregation hotspot [residues 306–311 (Figure 3D)] by using the flanking region 295–300 as a protective shield. Familial point mutations or alternative splicing can result in weakening of this secondary structural element, exposing the APR and promoting aggregation (Chen et al., 2019). Finally, for apolipoprotein apoA-I, involved in systemic amyloidosis, the aggregation hotspot (residues 14–22) is protected by a helix bundle formed at the N-terminus of the protein. Familial amyloidosis associated with disease-promoting mutations (such as G26R, W50R, F71Y, or L170P), all of which are located in regions flanking the amyloid hotspot, induce conformational changes that result in exposure of residues 14–22 and lead to amyloid formation (Adachi et al., 2014; Das et al., 2016).

The aggregation of globular proteins can also be affected by their flanking regions. One well-understood example is β_2_-microglobulin (β_2_m, a 99-residue protein with an Ig fold when natively folded) which is associated with the disease dialysis-related amyloidosis (DRA) (Gejyo et al., 1986). Approximately 30% of the molecules in fibrils of DRA patients is comprised of an N-terminally truncated variant missing the N-terminal six residues (Esposito et al., 2000). This deletion variant which is significantly more aggregation-prone than the full-length protein, is destabilized in its native state, and exhibits increased dynamics that facilitates amyloid formation (Chiti et al., 2001; Jahn et al., 2006; Eichner et al., 2011; Karamanos et al., 2019). An *in vitro* study that introduced point mutations into different β-strands of β_2_m further showed the importance of A- (residues 6–11) and G-strands (residues 91–94), both distant to the aggregation hotspot (Figure 3E). Amino acid substitutions in these strands (I7A, V9A, or V93A) induced aggregation by destabilizing the monomeric structure specifically in these regions, while similar mutations elsewhere in the sequence caused similar loss of thermodynamic stability, yet did not drive aggregation (Jones et al., 2003b). Hence local, rather than global, stability, is important in tailoring the aggregation of natively folded proteins, potentially because of the specific effects this has on fulfilling the aggregation-potential of a sequence’s APRs (Langenberg et al., 2020). In a similar vein, a rare mutation in β_2_m has recently been discovered in a French family that results in a different amyloid disease in which the variant protein (D76N) forms fibrils that deposit in the viscera without loss of renal function (Valleix et al., 2012). Importantly, the substituted amino acid (D76N) lies in a solvent exposed loop distant to the single APR in the protein (Figure 3E).

Other globular proteins have also been found to have flanking regions that are critically important for aggregation, including polyglutamine expansion (polyQ) proteins such as ataxin-3 (Saunders and Bottomley, 2009). This ∼40 kDa protein has a structured N-terminal protease domain (the Josephin domain) followed by an IDR which contains two (or sometimes three) ubiquitin interacting motifs (UIMs) and an expanded polyQ tract (Paulson, 2012). The aggregation mechanism of ataxin 3 *in vitro* has been shown to involve two kinetically resolved stages. In the first phase, the aggregation-prone Josephin domain self-associates into worm-like fibrils (that lack the cross-β structure of amyloid), with the slow formation of amyloid involving the polyQ tract occurring in a second phase (Ellisdon et al., 2006). Like all polyQ proteins, aggregation of ataxin 3 is critically dependent on the length of the polyQ tract. Importantly, the presence of a long (disease-causing) poly-glutamine tract changes the conformational dynamics of the Josephin domain, exposing the aggregation-prone N-terminal region and allowing self-association that results in aggregation (Gales et al., 2005; Scarff et al., 2015). Akin to the results for the mutation causing β_2_m aggregation discussed above, the polyQ tract is believed to diminish the stability of neighboring domains in ataxin 3, creating a local denaturing environment (Ignatova and Gierasch, 2006). The longer the polyQ sequence, the greater its effect in accelerating aggregation (Scarff et al., 2015).

In addition to intra- and inter-molecular homotypic association, interactions of flanking regions with other molecules/surfaces (heterotypic interactions) can also affect the kinetics of fibril formation (Sarell et al., 2013; Figure 4). For several amyloidogenic proteins, including IAPP, Aβ_40/42_ and αSyn, aggregation is accelerated in the presence of membranes (Terakawa et al., 2018). For example, the N-terminal region of αSyn binds to lipid bilayers (forming an α-helical structure) that leads to extensive surface-induced fibril growth (Fusco et al., 2014; Doherty et al., 2020; Table 1). For IAPP, the N-terminal 19 residues (which flank the core region of amyloid involving residues ∼15–37) bind to membranes and also become helical (Brender et al., 2008). Studies on an N-terminal fragment of IAPP involving residues 1–19 showed that membrane binding and disruption of the bilayer occur independently of fibril formation (Brender et al., 2008). Tau has also been shown to bind membranes, having functional as well as pathogenic effects where the lipid bilayer facilitates protein-protein interactions driving aggregation (Elbaum-Garfinkle et al., 2010). Another example where binding to membranes initiates a critical process can be found for PrP, a GPI anchor protein, as binding and insertion into lipid membranes (in the presence of RNA) initiates the conformational transition to a highly aggregation-prone form of PrP (Wang et al., 2010a). In addition, binding of PrP to anionic phospholipids is mediated by a flanking region outside of the fibril core and comprises the contiguous positively charged (residues 100–110) and “hydrophobic domain” regions (residues 111–134) (Wang et al., 2010b). Surface-induced aggregation is also important in the growing field of nanotechnology where nanoparticles coated with sugars, lipids or proteins, are widely used in drug delivery or diagnostics. These surfaces can also enhance protein aggregation. For example, β_2_m has been shown to aggregate more rapidly in the presence of copolymer particles, cerium oxide particles, quantum dots, and carbon nanotubes in a manner that is dependent on the surface area and surface modification (Linse et al., 2007). Fibril formation of IAPP is also enhanced in the presence of chiral silica nanoribbons (Faridi et al., 2018).

Fibril formation of one protein can also be affected by interactions with other amyloidogenic proteins. For example, αSyn aggregation is enhanced in the presence of Tau, which is biologically relevant since these two proteins are observed to co-aggregate in inclusions in brains from patients with Dementia with Lewy Bodies (DLB) (Colom-Cadena et al., 2013). Similarly, CsgA, a bacterial functional amyloid, also accelerates αSyn aggregation, possibly explaining clinical and epidemiological data that show an accumulation of aggregated αSyn first being found in olfactory epithelium or gastrointestinal tract, before spreading to the brain (Sampson et al., 2020). Aggregation assays with a C-terminally truncated variant of αSyn using PRE NMR experiments revealed that the C-terminal region of αSyn interacts with Tau (Dasari et al., 2019; Lu et al., 2020; Table 1). This region is also involved in ion binding, most importantly Ca^2+^, which drives fibril formation probably by changing the conformational dynamics of αSyn to a more extended form (Han et al., 2018). *In vivo* assays have shown that the native prion protein (PrP^C^) or protease-resistant isoform (PrP^Sc^) bind Tau with their N-terminal disordered segment (residue 23–90) (Han et al., 2006), and also bind to Aβ oligomers and αSyn fibrils, facilitating cell surface binding and cell-to-cell spreading (Laurén et al., 2009; Aulić et al., 2017). Additionally, single molecule Förster resonance energy transfer experiments (FRET) measuring the conformational ensemble of Tau have shown that heparin induces conformational changes that could be important in promoting amyloid formation (Elbaum-Garfinkle and Rhoades, 2012). These involve a loss of long-range contacts of the N- and C-terminal regions and a compaction of the aggregation-prone MBD (Elbaum-Garfinkle and Rhoades, 2012).

Finally, post-translational modifications including phosphorylation, ubiquitinoylation or acetylation are important regulatory modifications of amyloidogenic proteins that influence cellular mechanisms, such as protein degradation, signaling or protein-protein interactions, and also lead to misfolding and aggregation (Figure 4). Examples include the 441-residue protein Tau, in which 85 residues have been identified as phosphorylation sites mainly located in the aggregation hotspot that flanks the proline rich and C-terminal domains. Hyperphosphorylation of Tau is believed to trigger its dissociation from microtubules and to drive amyloid formation (Liu et al., 2007). However, recent studies on the four repeat region (K18) of Tau suggest an inhibitory effect of phosphorylation on fibril formation (Haj-Yahya et al., 2020). Finally, the protein huntingtin contains three lysines (K6, K9, K15) in its N-terminal 17-residue region which are often ubiquitinylated or SUMOylated, reducing toxicity by protease degradation or monomer stabilization, respectively (Ehrnhoefer et al., 2011).

### Roles of Flanking Regions in Protein Function

As proteins associated with amyloidosis have functional roles in their soluble states, aggregation can lead to a loss of function, as well as a gain of toxic function. For example, Tau (Table 1) is known to be present in six different isoforms in the central nervous system formed by alternative splicing processes. This results in deletions in the N-terminal region (45–103) or one of the four repeat regions, R2 (residues 275–305) (Himmler et al., 1989). The expression and translation levels of these isoforms are correlated with different developmental stages (Kosik et al., 1989), pointing to discrete functional roles of each. Indeed, Tau’s main function is to bind microtubules with its aggregation-prone MBD (residues 244–371) which induces assembly of microtubules. Although the MBD is primarily responsible for microtubule binding, its N-terminal and C-terminal flanking regions have been shown to modulate the conformation and accessibility of the MBD as part of its functional activity (Goode et al., 2000; Barbier et al., 2019). Further, the proline rich region (residues 151–243) (Table 1) also contributes to tubulin binding and its polymerization into microtubules (McKibben and Rhoades, 2019). Other amyloidogenic proteins such as αSyn are also considered to be microtubule-associated proteins (MAP). In this case much less is known, with current research suggesting a microtubule-polymerizing activity when αSyn binds tubulin via its C-terminal region (Alim et al., 2004), or supporting microtubule association and dynamics by binding probably via its N-terminal- and NAC-regions adopting a helical conformation (Cartelli et al., 2016). Oligomeric αSyn on the other hand has been shown to inhibit tubulin polymerization, resulting in cell death (Chen et al., 2007).

αSyn not only interacts with tubulin, but it also binds to many different proteins and molecules that are important for function (e.g., interaction with receptors to increase the neuronal levels of dopamine or inhibiting SNARE complex formation) and toxicity (e.g., the binding of αSyn to Parkin contributes to the pathophysiology of Parkinson’s disease) (Emamzadeh, 2016). The aggregation-prone NAC region of αSyn itself has been implicated in its function [e.g., binding to the dopamine receptor in neurons, regulating dopamine concentration (Lee et al., 2001)]. The ability of αSyn to bind to membranes is key to its physiological function of remodeling membrane vesicles within the presynaptic termini (Diao et al., 2013). *In vitro* NMR studies of αSyn:liposome interactions revealed that the N-terminal 25 residues trigger the interaction with the membrane by acting as an anchor motif, initializing binding of the whole N-terminal and NAC region (membrane sensor region, residues 26–98) with the formation of α-helical structure throughout this region (Fusco et al., 2014). Residues 26–98 are believed to modulate the affinity of αSyn for membranes and are crucial for its function in clustering synaptic vesicles (Fusco et al., 2016). Deleting or replacing residues 36–57 (the P1 and P2 regions discussed above), which flank the aggregation hotspot, showed an inhibition of the membrane remodeling activity, supporting a role of these flanking regions in liposome fusion (Doherty et al., 2020). A study from Cholak et al. (2020) suggests that the very N-terminal region (residues 1–14) of αSyn inserts into membranes to initiate membrane binding, with N-terminal acetylation of αSyn enhancing the lifetime of the membrane-bound state. In accord with the functional importance of these regions, αSyn shows higher sequence similarity to its two known homologs (βSyn and γSyn) in the N-terminal region compared with the C-terminal region (90% and 77% sequence identity in the N-terminal region and 36% and 1% sequence identity in the C-terminal region between αSyn and βSyn and αSyn and γSyn, respectively) (George, 2001). Membrane binding is not only involved in αSyn function, but it also represents a risk factor for αSyn aggregation and cytotoxicity, as association with membranes accelerates αSyn fibril formation (Galvagnion et al., 2016; Fusco et al., 2017). Binding to lipid bilayers via its N-terminal helical region has been suggested to be the pathological conformation of αSyn in yeast, causing slower cell growth and cell death (Newberry et al., 2020). Similarly, binding to mitochondrial membranes resulted in cytotoxicity due to enhanced formation of mitochondrial reactive oxygen species (ROS) and reduced ATP levels (Vicario et al., 2018; Ganjam et al., 2019).

The functional amyloid Orb2B is involved in forming long term memories (Keleman et al., 2007; Majumdar et al., 2012; Khan et al., 2015) by affecting translation within neurons (Krüttner et al., 2012) via its RNA binding domain (RBD). The RBD flanks a glutamine-rich region that drives the formation of fibrils whose formation activates translation (Hervas et al., 2020). TDP-43 is involved in maintaining mRNA stability, maturation and transport via specific RNA-recognition motifs (residues 104–262) (Prasad et al., 2019). For splicing, TDP-43 functions as a dimer stabilized by inter-molecular interactions in the N-terminal region, especially the N-terminal 10 residues (Shiina et al., 2010; Zhang et al., 2013; Afroz et al., 2017). These intermolecular interactions are also involved in initiating aggregation (Zhang et al., 2013), providing a further example of the tug-of-war between sequences involved in function that also enhance aggregation. TDP-43 also exhibits reversible liquid-liquid phase separation, a reversible process of de-mixing fluids into two distinct liquid-phases [reviewed by Alberti et al. (2019) and de Oliveira et al. (2019)] important for the formation of stress granules that store mRNA:protein complexes under cellular stress (e.g., oxidative or thermal stress) (McDonald et al., 2011; Sun and Chakrabartty, 2017). This function is mediated by the C-terminal prion-like domain of TDP-43 (Table 1). Substitution and deletion variants identified Trp334, Trp385, and Trp412 as important drivers of phase separation (Li H.R. et al., 2018). These residues flank the aggregation hotspot of TDP-43 [residues 288–346 (Figure 3B)] and only Trp334 is part of the fibril core in the dagger shaped polymorph (Cao et al., 2019). TDP-43 liquid droplets remain stable for only a short period of time (timescale of hours) before transforming into irreversible aggregates (Conicella et al., 2016). Using PRE NMR experiments, Conicella et al. (2016) identified residues 321–340 as those responsible for the crucial interactions for phase separation, by forming inter-molecular helix-helix self-assemblies that are disrupted by the ALS-associated mutations (A321G, Q331K, and M337V). Tau also undergoes liquid-liquid phase separation prior to the formation of gel-like and then amyloid-like aggregates (Wegmann et al., 2018). A detailed study of deletion variants revealed that electrostatic interactions between the N-terminal region (residues 1–117) and parts of the C-terminal domain (residues 118–402) drive phase separation [whereas residues in the microtubule binding region are thought to be important for amyloid formation (Elbaum-Garfinkle and Rhoades, 2012)]. Deleting either one of the former regions ablated droplet formation (Boyko et al., 2019). Other amyloid-associated proteins also undergo phase separation, at least *in vitro* (Elbaum-Garfinkle, 2019). For example, liquid-liquid phase separation of IAPP is catalyzed by the air-water-interface (Pytowski et al., 2020). Interestingly, Comparison with the non-fibrillogenic rat IAPP revealed that phase separation does not require the presence of the highly amyloidogenic region (residue 20–29), but hydrogelation and aggregation do (Pytowski et al., 2020). The prion-like functional amyloid Sup35 also undergoes phase separation, generating protein-specific environmental responses. In this protein, the N-terminal prion domain, as well as the flanking M-domain, have a benign role: promoting reversible phase separation and gelation in a pH dependent manner (Franzmann et al., 2018). Finally, αSyn has also been shown to form phase separated droplets which precede aggregation into amyloid *in vitro* and in cells (Ray et al., 2020) The cellular droplets later transform into perinuclear aggresomes, with the familial mutations and phosphorylation of Y39 as discussed above promoting liquid-liquid phase separation, as well as aggregation into amyloid.

## Regions Flanking the Structured Core of Amyloid Fibrils: The Importance of the Invisible Flanking Regions

**FIGURE 5:**
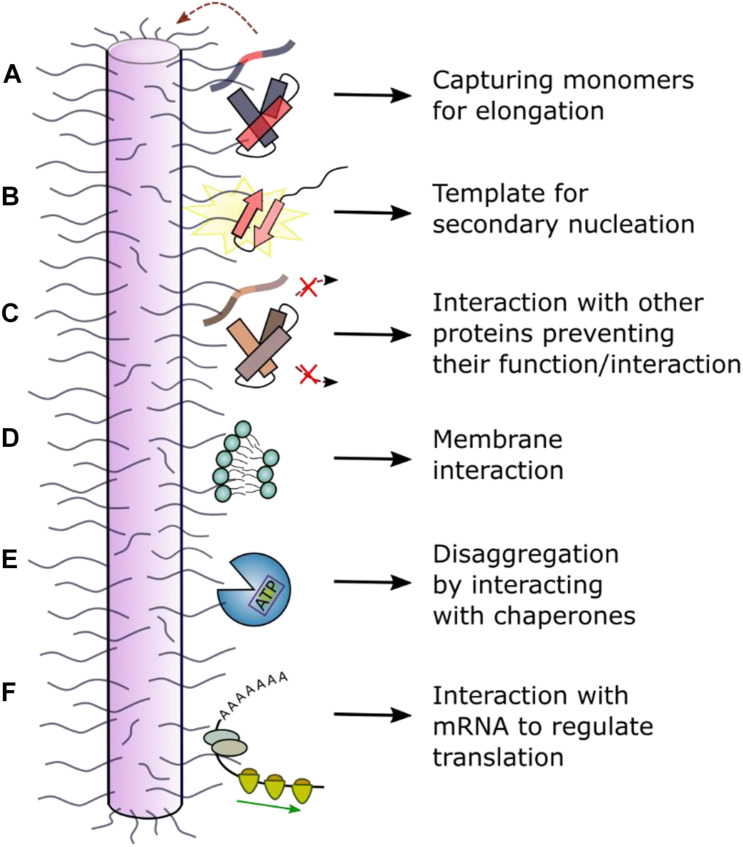
Schematic of interactions between the dynamically disordered regions of fibrils and other molecules. Dynamically disordered regions displayed on the surface of ordered amyloid fibrils can play roles in their function and cellular dysfunction. **(A)** The fibril “fuzzy coat” could capture amyloid precursors and facilitate their self-assembly into amyloid by **(B)** secondary nucleation or other molecular events. **(C)** Interaction with other (non-)amyloidogenic proteins could inhibit or alter their function. **(D)** Interaction with membranes can result in a toxic mechanism involving membrane disruption. **(E)** Interaction with chaperones can result in fibril depolymerization. **(F)** Interaction with RNA has been observed in a functional context for the protein CPEB (cytoplasmic polyadenylation element binding) which regulates long-term memory (Khan et al., 2015; Hervas et al., 2020). In other cases, disruption of cellular RNA could enhance phase separation and lead to cellular toxicity and/or dysfunction.

The biophysical and biochemical properties of each amyloid fibril polymorph and their effect on cells can differ dramatically. As amyloid formation is under kinetic control, changes in the protein sequence and/or the assembly conditions (*in vitro* or in cells) can affect the structure of the fibrils formed. Accordingly, different fibril structures for Tau isotypes have been observed to form in different amyloid diseases (Scheres et al., 2020). Interestingly, for αSyn amyloid, heterogeneity is also observed for fibrils from patients with Parkinson’s disease or Multiple system atrophy [detected using ssNMR and differences in the fluorescence emission spectra of extrinsic fluorophores (Strohäker et al., 2019)]. The ability to discriminate polymorphs by comparison of fluorescence emission spectra of some extrinsic fluorophores (e.g., Thioflavin T or Congo Red) when intercalated into cross-β fibril structures has been noted previously (reviewed by Bhattacharya and Mukhopadhyay, 2016). The differential binding affinity for some antibodies to oligomers and mature fibrils also highlights the structural conversion that has to occur for amyloid fibrils to form. For example, the Aβ_42_ antibody anti-Trx(Aβ15)_4_ recognizes a structural epitope of oligomers and fibrils but not of the monomer, with the same selectivity for other amyloidogenic proteins (Moretto et al., 2007). Consequently, it is perhaps not surprising that fibrils with different structures may induce different effects *in vivo*. Ferrari et al. (2020), for example, used a pull-down approach with FLAG-tagged Tau to show that different aggregation stages (monomer, oligomer, fibril) have altered reactivity with its cellular environment due to large conformational changes that occur when this natively unfolded protein self-assembles into a β-sheet rich fibrillary protein state. Given that the “fuzzy coat” of disordered peptide regions that flank the cores of amyloid fibrils can vary between fibril polymorphs (Gallardo et al., 2020b), the effect of these dynamic regions on the function, toxicity and further amplification of the fibrils themselves may also vary, perhaps rationalizing the epigenetic difference in disease development in individuals expressing the same aggregation-prone proteins. Below we discuss the importance of these structurally “invisible” flanking regions in amyloid formation and the cellular consequences of these regions in amyloid deposition (Figure 5).

### Fibril Elongation, Secondary Nucleation and Seeding

The extent of protein incorporation into insoluble fibrils is controlled by processes such as seeding (fragments broken off from fibrils, creating new fibril ends), elongation (the addition of monomers or oligomers onto fibril seeds) and secondary nucleation (creation of new nucleation sites on pre-existing fibril surfaces) (Michaels et al., 2018; Shvadchak et al., 2018; Scheidt et al., 2019). These kinetic events that drive amyloid formation could depend on the monomeric amyloid precursor, the structured amyloid core and the nature of the fibril “fuzzy coat.” For example, the flanking regions may be involved in the prion-like ability of αSyn to spread and seed further fibril growth (summarized by Longhena et al., 2017) as secretion of αSyn into the extracellular space occurs in association with membrane vesicles for which binding of the N-terminal region of αSyn is crucial (Emmanouilidou et al., 2010). The elongation rate may be accelerated as the large volume “fuzzy coat” may form transient interactions, “capturing” incoming monomeric protein molecules (Tompa, 2009; Figure 5A). This model is supported by the example of the human Prion Protein (PrP), in which increased numbers of an octapeptide Cu^2+^-binding motif in the N-terminal IDR (which flanks the folded C-terminal prion domain) promotes fibril formation and disease development (Goldfarb et al., 1991). Additionally, the fibril surface might have a key role in surface-induced secondary nucleation (Figure 5B). This is because the surfaces of all but one (Gremer et al., 2017) amyloid fibril structures solved to date are decorated by potentially large region of dynamically disordered protein flanking regions. Secondary nucleation for αSyn and other amyloid proteins, including Aβ and IAPP, is strongly dependent on pH (Gaspar et al., 2017). This could be explained by fact that the pH affects the dynamics of the solvent exposed flanking regions, as well as the charge along the ordered fibril core (Wegmann et al., 2013). The importance of flanking regions for these processes is illustrated by co-incubation of αSyn and βSyn. NMR analysis of the fibrils formed show increased dynamics in the N-terminal region compared with pure αSyn fibrils, whilst the core structure is unchanged. These co-incubated fibrils exhibited a reduced seeding capacity (Yang et al., 2019). However, the precise molecular mechanism(s) of the elongation and secondary nucleation processes, and how they alter with changes in sequence and fibril structure is not currently understood in detail.

### Interaction With Other Amyloidogenic Proteins

Amyloid fibrils can interact with other amyloidogenic precursors and alter their amyloid potential (Figure 5C). αSyn fibrils, for instance, have been shown to bind Tau monomers via the αSyn acidic C-terminal region which is dynamically disordered in all fibril structures determined to date (Guerrero-Ferreira et al., 2020; Schweighauser et al., 2020). Since binding of Tau stabilizes microtubules, αSyn fibrils indirectly affect microtubule stability by removing Tau from microtubule surfaces resulting in neuronal dysfunction (Oikawa et al., 2016). Interaction of αSyn fibrils with Tau monomers further induces a conformational change in Tau, promoting its subsequent assembly into Tau amyloid structures (Oikawa et al., 2016). As reviewed by Luo et al. (2016), Aβ interacts with at least 10 other disease-related amyloidogenic proteins (e.g., IAPP, Tau, αSyn). For example, aggregation of Aβ_40/42_ is inhibited by cystatin C (Sastre et al., 2004), a protein which also colocalizes with Aβ_40/42_ in brain amyloid deposits. ELISA assays with an antibody targeting the N-terminal end of Aβ (residues 1–17) defined the binding site to be the first 17 residues, since binding was abolished in the presence of the antibody (Sastre et al., 2004). Aβ_40/42_ aggregation is also inhibited in the presence of TTR tetramers. NMR experiments revealed the interaction site to be between the thyroxine binding pocket of the TTR tetramer and Aβ residues 18–21 (Li et al., 2013). Finally, amyloid proteins have been observed to interact with other proteins in the context of cross-seeding. For example, pre-formed fibrils of IAPP have been shown to cross-seed Aβ_40_ monomers, accelerating fibril growth (Moreno-Gonzalez et al., 2017). The exact binding site is not known, but the two proteins have been shown to co-localize in disease (Moreno-Gonzalez et al., 2017).

### Membrane Binding

Although many studies have shown that oligomers can be cytotoxic and possibly a major culprit of amyloid diseases (Fändrich, 2012; Verma et al., 2015; Fusco et al., 2017; Karamanos et al., 2019), the surface-induced fibril growth process on membranes (Engel et al., 2008; Qiang et al., 2015) and the interaction of mature fibrils with membranes (Figure 5D) have both been shown to disrupt cellular function and homeostasis (Martins et al., 2008; Xue et al., 2009; Pieri et al., 2012). *In vitro* experiments on αSyn have shown that its kinetics of aggregation are strongly affected by the presence of liposomes, highlighting the important role of lipid bilayers for fibril formation (Galvagnion et al., 2015). αSyn fibrils (and oligomers) bind negative, but not neutrally charged, liposomes (Grey et al., 2011; Pieri et al., 2012), pointing to an interaction with the N-terminal positively charged flanking region, which is dynamically disordered in the fibrillar state, similar to the interaction that is observed in the monomeric state. Striking work from Bäuerlein et al. (2017) used cryo-electron tomography *in situ* to show that polyQ fibrils (from huntingtin-exon 1) interact with membranes of the endoplasmic reticulum, changing the dynamics and structural organization of the organelle. These studies also showed that the sides of the fibril, as well as fibril ends, interact with membranes, although which regions of the fibril are involved in the interaction (the core or dynamic sequences) is not currently known. Similar vesicle membrane disruption was observed *in vitro* for β_2_m fibrils, with the majority of fibrils binding via their ends (Milanesi et al., 2012). Interestingly, the most severe membrane damage was observed for membranes containing the lipid BMP [bis(monoacylglycero)phosphate] which is enriched in lysosomal membranes, rationalizing the role of this organelle in the etiology of many amyloid diseases (Goodchild et al., 2014).

### Interaction With Chaperones

Amyloid fibrils have been shown to interact with chaperones in different contexts (Figure 5E) of which neuroprotection is one of the foremost (Kannaian et al., 2019). Chaperone binding functions as an inhibitor for primary nucleation (when binding monomers), as well as for secondary nucleation and elongation, and some chaperone systems can even induce disaggregation of fibrils in the presence of ATP (Kannaian et al., 2019). Chaperones such as αB-crystallin have been shown to bind along the length of the fibril surface, perturbing secondary nucleation and elongation for both Aβ_40_/Aβ_42_ and αSyn (Waudby et al., 2010; Shammas et al., 2011). However, despite the authors revealing fascinating images of the chaperone *in situ* using immunogold labeling and immunoelectron microscopy, the exact binding site could not be determined due to the low resolution of this technique. In the case of Hsp27, which binds on the surface of αSyn fibrils causing decreased cytotoxicity and inhibited elongation, total internal reflection fluorescence (TIRF)-based imaging suggested that Hsp27 preferentially binds to hydrophobic patches along the fibril surface (Cox et al., 2018). These hydrophobic motifs involved in the interaction could involve the dynamic regions [residues 1–6 or 36–42 (Figure 3A)] as well as the fibril core itself. A higher resolution analysis, e.g., using HX methods, or more directly using ssNMR, is required to identify the exact chaperone binding sites.

Given the high thermodynamic stability of fibrils, it is perhaps a remarkable feat that chaperones can induce their depolymerization. For αSyn fibrils, for example, Bukau and coworkers have shown that the human chaperone Hsc70, specifically in complex with DNAJB1 (Hsp40 family) and Apg2 is a hsp110 family member (but not other co-chaperones from these families), induces ATP-dependent fibril fragmentation and depolymerization (Gao et al., 2015). Experiments using deletion variants allowed the interaction site of the fibrils with the chaperones to be identified, involving residues 1–30 and 111–140: the flanking regions of the αSyn amyloid core. A similar disaggregase activity has been observed for the Lipocalin-type Prostaglandin D synthase (L-PGDS) and Aβ_40_ fibrils (Kannaian et al., 2019). L-PGDS binds to the central region of the Aβ_40_ sequence (G25–G29). As this sequence forms a bend connecting two β-strands (Petkova et al., 2002) it is part of the structured fibril core (Figure 3C), yet is solvent accessible. L-PGDS fulfils its chaperone activity without ATP consumption or any co-chaperones. Also, the protease HTRA1^S 328A^ can act as a chaperone and disassemble pre-formed 4R Tau filaments (Poepsel et al., 2015). Such observations may provide exciting new strategies to reduce the fibril load in amyloid diseases involving intracellular amyloid deposition.

### Interaction With RNA

Several amyloidogenic proteins and prions have been identified in the context of RNA-modulating functions (Nizhnikov et al., 2016). Usually, as in the case of TDP-43, the native monomeric protein is involved in binding the RNA (see above). However, fibrils can also interact with RNA, as shown for the functional amyloid CPEB, that is involved in long term memory formation (Keleman et al., 2007; Majumdar et al., 2012; Khan et al., 2015). A study on the *Drosophila melanogaster* CPEB homolog, Orb2B, demonstrated that RNA binding to the monomeric protein represses protein translation of some genes, whilst binding to oligomeric states and fibrils activates protein production by stabilizing and elongating the poly(A) tail of mRNA in neurons in complex with other proteins (e.g., CG4612) (Khan et al., 2015; Hervas et al., 2020; Figure 5F). Translation activation changes the synthesis of specific synaptic proteins involved in memory formation. A high resolution cryoEM fibril structure demonstrated that only a small part of the Orb2 sequence (residues 176–206) forms the fibril core (Figure 3F). The RNA-recognition motif and protein interaction domain are located in the long flanking regions of this 704 residue protein (Hervas et al., 2020).

## Discussion

In this review we have discussed the roles of regions that flank the APR sequences in monomeric amyloid precursors, and the role of the dynamically disordered regions that flank the structured core of amyloid fibrils in the interaction with other molecules and how this impacts cellular (dys)function. The importance of APRs is well recognized and their prediction using various computational tools is now straightforward. How the sequences that flank the APRs affect the kinetics and mechanisms of fibril growth, the structures of fibrils that ultimately form, and the extent and chemical identity of the dynamically disordered “fuzzy coat” regions of fibrils is still not well understood. This understanding is important as flanking regions play vital roles in the formation and interaction of amyloid with the cell and hence in disease.

In several native amyloid precursors, whether initially disordered or structured, regions surrounding the APRs or aggregation hotspot have been shown to be crucial for modulating amyloid formation. Intrinsic interactions in IDPs, including the APR flanking regions, can either promote or disfavor aggregation, by altering the conformational landscape of the IDP and “switching” amyloid formation on or off, as clearly shown for αSyn, Aβ, and Tau (Elbaum-Garfinkle and Rhoades, 2012; Stephens et al., 2019; Doherty et al., 2020). Also, for initially natively folded proteins, such as β_2_m, regions that flank the APRs often stabilize the native conformation. Mutations or truncations in these regions can consequently release the aggregation potential of the APR by locally destabilizing the protein fold leading to fibril formation (Langenberg et al., 2020). Advanced biophysical methods, such as NMR PRE experiments and single molecule FRET analysis can be used to identify transient long-range interactions in IDPs and IDRs in all-residue, if not all-atom, detail. Complemented by molecular dynamics simulations and experiments using deletion or substitution variants, these approaches have helped to identify the role of these “master controller” (Doherty et al., 2020) motifs for amyloid formation and provide targets to develop new strategies to combat amyloid formation and disease (Mirecka et al., 2014; Agerschou et al., 2019; Chen et al., 2019).

In addition to homotypic intra- and inter-molecular events, interactions with other molecules can be vital for amyloid function and pathology. Determining the interaction site of an amyloidogenic protein, or an amyloid fibril, with other molecules can be challenging, given the dynamic nature of the proteins involved. However, NMR experiments using PREs or chemical shifts, or binding assays with deletion variants can enable the identification of the residues that are involved in these binding processes. Such experiments can be used to clarify whether the aggregation hotspot or its flanking regions are required for the interaction, and potentially provide an evolutionary explanation for the development of such high-risk sequences.

Proteins undergo dramatic conformational changes on the pathway from initial precursor to amyloid states. More focus on these structural changes and which parts of the protein sequence drive these transformations might further demonstrate a crucial role of flanking regions in the amyloid cascade. Flanking regions of the fibril core are relatively straightforward to identify (e.g., using HX or protease protection experiments) but are especially hard to analyze structurally since their dynamic properties displayed on a static high molecular weight fibrillar particle provide significant experimental and computational challenges. All high-resolution structural information on amyloid fibrils formed from intact proteins [rather than short peptides and peptide fragments (Guenther et al., 2018; Rodriguez et al., 2015)] has been gained using ssNMR or cryoEM (Fitzpatrick and Saibil, 2019; Gallardo et al., 2020b; Guerrero-Ferreira et al., 2020; Scheres et al., 2020). These techniques, however, cannot provide atomic resolution information about the structure of the flexible regions which can comprise the large majority of the protein sequence in some amyloid states (Figure 3; Gallardo et al., 2020b). Nevertheless, some studies have shown the importance of the amyloid “fuzzy coat” for interactions with other amyloid precursors, cellular membranes, RNA and chaperones (Figures 4, 5). Assays with protease-treated fibrils, where the “fuzzy coat” is shaved off, would allow a better understanding of which part of the fibril is involved in the binding process and how the cellular consequences of fibril formation depend on the dynamically disordered regions displayed on the cross-β amyloid fold.

Fibril polymorphism has been shown to be responsible for the development of different diseases caused by the same protein [e.g., αSyn causing Parkinson’s disease or Multiple System Atrophy or Dementia with Lewy Bodies (Shahnawaz et al., 2020) or Tau causing Alzheimer’s disease, Pick’s disease, chronic traumatic encephalitis, and corticobasal degeneration (Fitzpatrick et al., 2017; Falcon et al., 2018; Scheres et al., 2020)]. Polymorphs, so far, have been defined by a fibril possessing a different core: but the flanking regions to the core could also be different in fibrils with the same (or similar) core structures. This adds a further, currently unexplored, dimension to amyloid polymorphism and its consequences for disease, since the dynamically disordered regions could have different interactions with cellular components (Wegmann et al., 2013). A more detailed analysis of the extent of conformational fluctuation of these amyloid flanking regions, for instance using EPR, cross-linking, or other techniques able to tackle dynamic heterogeneity, in the future might reveal “polymorphism” in the “fuzzy coat” and how this is related to the development of disease.

It should not be forgotten that flanking regions can have very different lengths. In the case of Aβ_40/42_, (nearly) the whole protein forms the fibril core (Figure 3C) (Lu et al., 2013; Gremer et al., 2017), whilst in other proteins, e.g., Orb2, flanking regions >500 residues in length are observed (Figure 3F; Hervas et al., 2020). Tompa (2009) hypothesized that the longer the dynamically disordered region, the more likely it can interact with other molecules by a “fly fishing mechanism.” This might explain why functional amyloid fibrils such as Orb2 have long flanking regions able to interact with other proteins, RNA or surfaces, whilst pathological amyloid fibrils, the sequences of which have not evolved for functional reasons, may present shorter flanking regions.

## Conclusion

In summary, this review highlights the crucial role of regions that flank the APRs in amyloidogenic protein sequences and the dynamic regions that flank the amyloid fibril core for function, fibril formation and cellular dysfunction for a few example proteins. While beautiful fibril structures are now emerging from cryoEM and ssNMR studies, the often poorly visible, but functionally important flanking regions must not be forgotten. Focusing more on these regions with a broad range of biophysical and cellular techniques might help to gain a better understanding of the molecular mechanism of fibril formation and to identify new targets for drug development that do not involve the ordered amyloid core and the aggregation hotspots.

## Author Contributions

SMU, DJB, and SER wrote the manuscript. All authors contributed to the article and approved the submitted version.

## Conflict of Interest

The authors declare that the research was conducted in the absence of any commercial or financial relationships that could be construed as a potential conflict of interest.
